# Dynamics of grain boundary premelting

**DOI:** 10.1038/s41598-020-77863-9

**Published:** 2020-12-03

**Authors:** M. Torabi Rad, G. Boussinot, M. Apel

**Affiliations:** Access e.V., Intzestr. 5, 52072 Aachen, Germany

**Keywords:** Phase transitions and critical phenomena, Computational methods

## Abstract

The mechanical strength of a polycrystalline material can be drastically weakened by a phenomenon known as grain boundary (GB) premelting that takes place, owing to the so-called disjoining potential, when the dry GB free energy $\sigma _{gb}$ exceeds twice the free energy of the solid–liquid interface $\sigma _{sl}$. While previous studies of GB premelting are all limited to equilibrium conditions, we use a multi-phase field model to analyze premelting dynamics by simulating the steady-state growth of a liquid layer along a dry GB in an insulated channel and the evolution of a pre-melted polycrystalline microstructure. In both cases, our results reveal the crucial influence of the disjoining potential. A dry GB transforms into a pre-melted state for a grain-size-dependent temperature interval around $T_m$, such that a critical overheating of the dry GBs over $T_m$ should be exceeded for the classical melting process to take place, the liquid layer to achieve a macroscopic width, and the disjoining potential to vanish. Our simulations suggest a steady-state velocity for this transformation proportional to $\sigma _{gb} -2 \sigma _{sl}$. Concerning the poly-crystalline evolution, we find unusual grain morphologies and dynamics, deriving from the existence of a pre-melted polycrystalline equilibrium that we evidence. We are then able to identify the regime in which, due to the separation of the involved length scales, the dynamics corresponds to the same curvature-driven dynamics as for dry GBs, but with enhanced mobility.

## Introduction

In a polycrystalline material at a temperature well below the bulk melting temperature $T_m$, the disordered region at a Grain Boundary (GB) is only a few atomic distances wide. As the temperature increases towards $T_m$, however, GBs may turn into a liquid-like, thermodynamically stable thin (i.e., nanoscale) film. This order-disorder transition is termed GB premelting^1^. Premelting may be observed not only at the GBs but also on the surfaces^2,3^ and, due to its physically-rich nature and important consequences in wide range of applications, has interested scientists from not only chemistry and physics domains, but also from domains such as earth science^4^, fluid mechanics^5^, and, interestingly, biology^6^.

GB premelting has implications in, for example, general metallic systems^1^, steel^7^, and ceramics^8^. It increases GB diffusivity and mobility^9,10^, and can drastically influence the macroscopic properties of a material. For example, it can reduce the resistance to shear stresses^11,12^, which can result in material failure in high-temperature applications. GB premelting is also linked to cracking during late-stage solidification of metallic alloys^13,14^, to liquid metal embrittlement^15^ and in general influences grain growth, for example in situations such as brazing. Despite these implications, GB premelting is still not fully understood one reason being the lack of abundant experimental studies (see^16^ and references therein), which is mainly due to the challenges in observing internal material interfaces such as GBs. Another reason is that the existing experimental evidence for pure materials is controversial^12^ due to a fair criticism that the conclusions could have been influenced by trace impurities^17^.

In the literature, basic thermodynamics are invoked to suggest that pre-melted GBs form because solid–liquid interfaces may repulse each other and form a liquid-like layer to decrease the free energy of the system^12^. Generically, the energy $\sigma _{gb}$ of a low-angle GB is small and proportional to the misorientation between the adjacent grains^18^, while $\sigma _{gb}$ is higher for high-angle GBs. Moreover, $\sigma _{gb}$ may also be influenced by the presence of GB adsorption. Then, when $\sigma _{gb}$ is larger than twice the energy of well-separated solid–liquid interfaces $\sigma _{sl}$, the system can lower its free energy by replacing the dry GB with two solid–liquid interfaces and a liquid layer between them. This is quite similar to the situation where a solid in contact with vapor starts to melt at its surface when it is energetically favorable to replace the solid/vapor interface by a solid/liquid interface and a liquid/vapor interface^19^.

The thickness of the liquid layer at a pre-melted GB is determined by the competition, as the distance between the solid–liquid interfaces varies, between the changes in bulk and interfacial free energies. Theoretically, the variation of interface energy is provided by a so-called disjoining potential, that describes energetically the deviation of the atomic structure in the thin liquid layer from the one in the bulk liquid and the interaction between the solid–liquid interfaces. This potential allows the interface energy to be interpolated continuously between $\sigma _{gb}$, at vanishing distance between the solid–liquid interfaces, and $2\sigma _{sl}$ at infinite distance^20^.

Several computational methods were employed in order to study premelting. Using Lennard–Jones or embedded-atoms-method potentials, Molecular Dynamics^21–23^ and Monte–Carlo simulations^24,25^ were performed on the atomistic level. Phase-field or diffuse-interface methods were also used. In Refs.^26–28^, one order parameter discriminates between solid and liquid, and one orientation field describes crystallographic orientation in the solid phase. In Refs.^16,29,30^, a multi-phase-field model is used, in which the liquid and each grain are characterized by a separate field. More recently, the phase-field-crystal method was also employed^12,31–33^, giving also some hints on the microscopic mechanisms of premelting.

In general, premelting under equilibrium conditions is mainly controlled by the dependence of the disjoining potential on the distance between the two solid–liquid interfaces and by the variation of this dependence with temperature. In the simplest case, the disjoining potential is monotonous for any temperature, and the equilibrium width of the liquid layer varies continuously with the temperature. As soon as the disjoining potential becomes non-monotonous, the scenario becomes more complicated. In particular, so-called “thin-to-thick” first-order transitions may occur when the disjoining potential corresponds to damped oscillations^25^, with a coexistence in a certain temperature interval of several equilibrium widths.

The above studies have resulted in valuable insights into the physically-rich nature of GB premelting. They have, however, to the best of our knowledge, considered only premelting under equilibrium conditions. In this article, we are interested in the dynamics of premelting, i.e., how a liquid layer penetrates along dry GBs driving the transformation of the latter into a pre-melted equilibrium. More generally, we consider the situation where a fully-solid and low-temperature polycrystalline structure is brought to a higher temperature close to $T_m$. Fundamental open questions include the effect of the grain size on the dynamical behavior of a pre-melted layer, the dependence of the penetration velocity on the GB and solid/liquid interface energies, the shape of the liquid layer around its tip, and the influence of the initial temperature on the final structure, i.e. the product of the transformation. We address those questions using the multi-phase-field model with obstacle potentials that is implemented in MICRESS^34^. While the phase-field-crystal model and molecular dynamics techniques have been used for studying premelting in equilibrium conditions, the phase field model is used here to resolve the length scale associated with the diffusion field, too large to be addressed with the former methods. We first present the solution for the pre-melted equilibrium that this model yields. Assuming a steady-state growth of the liquid layer along the dry GB, we analyze the consequences of energy conservation on the equilibrium state that develops as a product of the transformation. We then present simulations that focus on the triple junction and on the dependence of the growth velocity on the interface energies. Next, we present the simulation of the evolution of a polycrystalline structure.

## Theoretical phase-field analysis

Phase field models describe the evolution of continuous fields that may represent for example the local temperature, chemical composition or fraction of each phase under consideration. In the latter case, the fields are called ‘phase fields’ and their variations represent interfaces, for example between two grains or between a solid and a liquid. They may also represent triple junctions when three fields are involved, or even higher order junctions.

The premelting of a GB is linked to the existence of a so-called disjoining potential between solid/liquid interfaces that are separated by small enough distances. This interaction between solid/liquid interfaces describes the fact that the surface energy may be considered as going continuously from the value of the GB energy $\sigma _{gb}$ at vanishing distance (i.e. when the two solids grains are in contact), to twice the value of the solid/liquid interface energy $\sigma _{sl}$ at infinite distance. A certain variety of premelting scenarios can be expected depending on the shape of the disjoining potential^27,28^. When the latter varies monotonically with the distance, the scenario is however rather simple and premelting occurs when $\sigma _{gb} - 2\sigma _{sl} > 0$, with a unique solution linking the width of the liquid layer to the temperature at equilibrium.

In the phase field model, the disjoining potential is related to the overlap of the phase fields of the two grains. There are mainly two ways of describing the dynamics of the phase fields. In the first one with so-called double well potentials, the phase fields exhibits spatial variations of an hyperbolic tangent type, with an exponential convergence to the value 0 or 1. In this frame, it was shown^29^ that premelting is not properly reproduced, especially because, although being able to be repulsive at short distances, the disjoining potential is always attractive at large ones, regardless of the ratio of GB to solid/liquid energies. In the second one, which we use in this article, with so-called obstacle potentials, the phase fields spatial variations are strictly restricted to a finite length scale and the fields reach exactly their value 0 or 1 at a finite distance from the center of the interface. The corresponding evolution equations are presented in the “Methods”. It was shown (see Fig. 10 in Ref.^30^) that such a model provides, regardless of the distance, a repulsive (attractive) interaction when $\sigma _{gb} - 2 \sigma _{sl} > 0 \;(<0)$. This model thus reproduces premelting in a manner quite similar to a sharp interface description using a monotonous disjoining potential, for example a decaying exponential as in Ref.^35^.

### Premelting equilibrium

The pre-melted equilibrium reproduced by the multi-phase field model with obstacle potentials, was analyzed in Ref.^30^, and we give here a brief overview of that analysis. The problem is one-dimensional and in the following all lengths are expressed in units of $\eta /(2\pi )$, where $\eta$ is the width of the interface, an intrinsic property of the phase field model. Three phase-fields $\phi _1(x), \phi _2(x)$ and $\phi _3(x)$ are defined so as to describe the existence of three phases: two solid grains (solid 1 with $\phi _1$ and solid 2 with $\phi _2$) and the liquid ($\phi _3$). The sum of these phase fields equals unity at any position in space:

1 $$\begin{aligned} \phi _1(x)+\phi _2(x)+\phi _3(x)=1. \end{aligned}$$

The problem is invariant in exchanging solid 1 and solid 2, so that the fields obey some symmetry when $x=0$ is chosen as the center of the liquid film:

2 $$\begin{aligned} \phi _1(x) = \phi _2(-x), \end{aligned}$$

3 $$\begin{aligned} \phi _3(x)=\phi _3(-x). \end{aligned}$$

**Figure 1:**
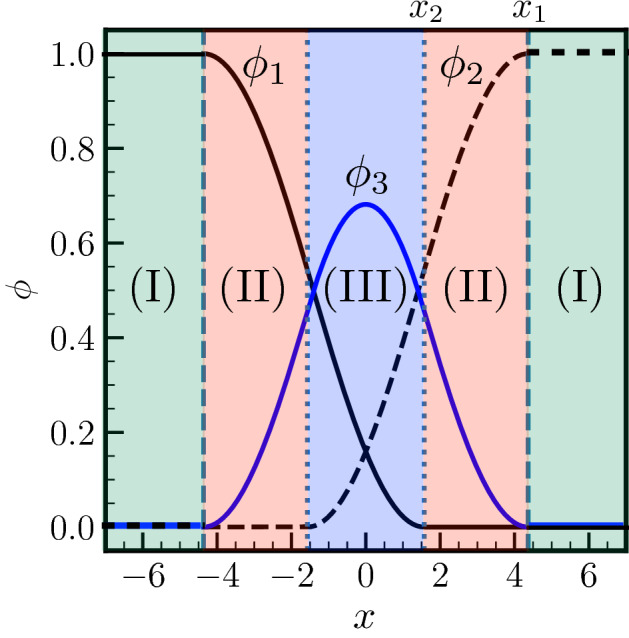
Phase fields profiles for the pre-melted equilibrium corresponding to a liquid layer, represented by $\phi _3(x)$, between grain 1, represented by $\phi _1(x)$, and grain 2, represented by $\phi _2(x)$. The sum rule $\sum _i \phi _i(x)=1$ holds. In region (I) at negative (positive) *x*, grain 1 (grain 2) is in its thermodynamically stable bulk state with $\phi _1=1$ ($\phi _2=1$). The liquid layer is present with $\phi _3(x) \ne 0$ in regions (II) and (III) (see text for more details). The degree of disorder in the liquid layer $\phi _3$ is characteristic of premelting with $\phi _3 <1$.

Three regions may then be defined, depending on the local number of phase fields that assume a non-vanishing value, as depicted in Fig. 1 in different colors. The black solid line represents $\phi _1$, the black dashed line represents $\phi _2$ and the blue line represents $\phi _3$. Corresponding to region (I), we have $\phi _1(-\infty<x<-x_1)=1$ and $\phi _2(x_1<x<+\infty )=1$. Corresponding to region (II), we have $\phi _3 (-x_1<x<-x_2) = 1 - \phi _1(x)$, which means that only solid 1 and the liquid are present at $-x_1<x<-x_2$ ($\phi _2=0$), and only solid 2 and the liquid are present at $x_2<x<x_1$ ($\phi _1=0$). In region (III), all three phase fields are non-vanishing. It is important to note that

4 $$\begin{aligned} x_1 + x_2 = 2\pi . \end{aligned}$$

**Figure 2:**
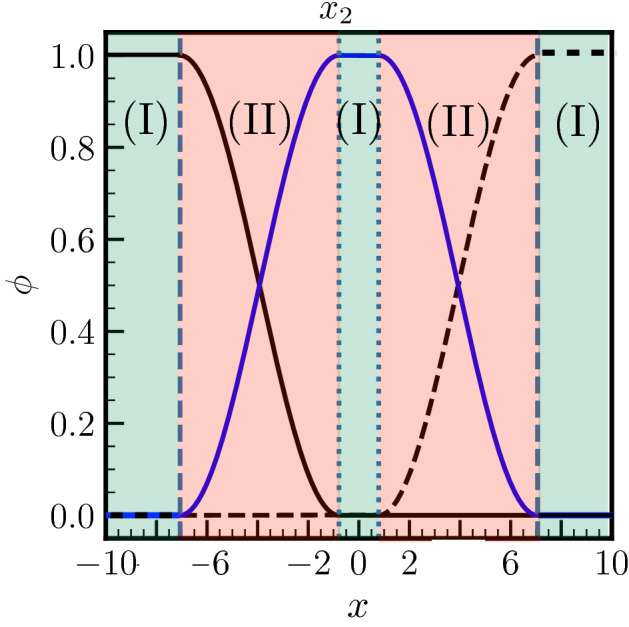
Phase fields profiles for a macroscopic solid–liquid equilibrium. The degree of disorder in the region (I) around $x=0$ is characteristic of bulk liquid with $\phi _3=1$ (see text for more details).

It can be seen within this frame that $\phi _3$ may not reach unity. We are thus in the case of a pre-melted system for which a liquid layer presenting a certain degree of crystalline order ($\phi _3 \ne 1$) separates two solid grains. The larger is $\phi _3$ at the center of the liquid film, i.e. $\phi _3(x=0)$, the larger is the disorder of this pre-melted GB. When $x_2$ increases (and $x_1$ decreases consequently owing to $x_1+x_2=2\pi$), the level of disorder in the liquid layer decreases ($\phi _3$ decreases), and when $x_2=x_1=\pi$, $\phi _3$ vanishes identically throughout the whole system, i.e. the GB is dry. On the other hand, when $x_2$ decreases, i.e. $x_1$ increases, the liquid phase field $\phi _3$ increases, and when $x_2=0$, i.e. $x_1 = 2\pi$, the level of disorder at the center of the liquid layer is characteristic of the bulk liquid, i.e. $\phi _3(x=0)=1$. When $x_2$ decreases further, i.e. when $x_2<0$, region (III) disappears, the two solids are no more interacting via the disjoining potential ($\phi _1$ and $\phi _2$ are no more overlapping), and a macroscopic liquid film having the structure of the bulk liquid, with $\phi _3(x_2<x<-x_2)=1$, is then in equilibrium with the two solids. This situation is depicted in Fig. 2.

In the following we denote as a pre-melted GB the situation where $0<\phi _3(0)<1$ and a melted GB when $\phi _3(0)=1$. Thus premelting occurs in a finite range of $x_2$, i.e. $0<x_2<\pi$. This is in contrast to a sharp interface description where the disjoining potential exhibits a exponential decay with the distance between the solid–liquid interfaces. Indeed, in this case, the magnitude of the disjoining forces never strictly vanishes, and the width of the liquid layer at equilibrium diverges logarithmically when the temperature *T* approaches from below the bulk melting temperature $T_m$. Here, in the phase field model, $0<T_m-T \rightarrow 0$ means $0<x_2 \rightarrow 0$, and $x_2<0$ corresponds to $T=T_m$.

The analytical solution of the phase field equations for the pre-melted equilibrium, i.e. for $0<x_2<\pi$, reads as follows (see Ref.^30^ for more details):

5 $$\begin{aligned} \phi _1(x)= {\left\{ \begin{array}{ll} 1, &{} \quad x< -x_1\\ \phi _1^{(\mathrm{II})}(x), &{} \quad -x_1< x< -x_2\\ \phi _1^{(\mathrm{III})}(x), &{} \quad -x_2< x< x_2\\ 0 &{} \quad x_2< x\\ \end{array}\right. }; \quad \phi _2(x)= \phi _1(-x) ; \quad \phi _3(x)=1-\phi _1(x)-\phi _2(x) ; \quad 0<x_2<\pi \end{aligned}$$

and

6 $$\begin{aligned} \phi _1^{(\mathrm{II})} (x)= & {} \frac{1+\Delta }{2} + \frac{1-\Delta }{2} \cos \left( \frac{x + x_1}{2} \right) , \end{aligned}$$

7 $$\begin{aligned} \phi _1^{(\mathrm{III})} (x)= & {} \frac{1+\Delta }{4 - \sigma ^*} \left[ 1 - \cos \left( \frac{x - x_2}{2} \right) \right] , \end{aligned}$$

with

8 $$\begin{aligned} \Delta = L \eta (T-T_m)/(4\sigma _{sl} T_m) \end{aligned}$$

and *L* corresponds to the latent heat of fusion related to the entropy difference between solid and liquid at $T_m$. The dimensionless quantity $\sigma ^* = \sigma _{gb}/\sigma _{sl}$ is a measure of the tendency for the GB to spontaneously melt and premelting occurs when $\sigma ^*>2$. It can be seen that within this model $\sigma ^*$ should remain smaller than 4 for premelting to be described in the terms presented above.

As mentioned above, the level of disorder of the pre-melted GB may be quantified by $\phi _3(x=0) = 1 - \phi _1(x=0) - \phi _2(x=0) = 1 - 2 \phi _1^{(\mathrm{III})} (x=0)$, i.e.

9 $$\begin{aligned} \phi _3(0) = 1 - 2 \; \frac{1+\Delta }{4 - \sigma ^*} \left[ 1 - \cos \frac{x_2}{2} \right] . \end{aligned}$$

For a given $\Delta$, while $x_1 = 2\pi - x_2$ (owing to $x_1+x_2=2\pi$), $x_2$ is found as the solution of

10 $$\begin{aligned} \Delta = 1 + \frac{2}{\beta } \left( 1- \sqrt{1+\beta } \right) , \end{aligned}$$

11 $$\begin{aligned} \beta = - \frac{(\sigma ^*-2) (1 - \cos x_2 ) }{ (\sigma ^* - 2)^2/4 +1 - (\sigma ^*-2) \cos x_2}. \end{aligned}$$

This explicit relation between the temperature $\Delta$ and $x_2$, that was not given in Ref.^30^, will be used in the following paragraph where we study energy conservation during steady-state propagation of the liquid film along the GB.

Let us now consider the two transitions that are described above. For $x_2=x_1=\pi$, corresponding to $\phi _3(x)=0$, we have $\Delta = - (\sigma ^* - 2)/2$. This temperature corresponds to the onset of premelting, i.e. it is the minimum temperature at which premelting can be expected. Note that in the neighborhood of this transition, i.e. when $x_1-x_2 \ll 1$, numerics are tedious since the grid spacing should much smaller than $x_1-x_2$. For $x_2 = 0$, one has $\Delta =0$, corresponding to the melting temperature, i.e. the onset of melting. The level of disorder at the center of the liquid film is then characteristic of the bulk liquid, i.e. $\phi _3(0) = 1$. It should be noted that the onset of premelting approaches the onset of melting when $\sigma ^*$ approaches 2. Then, $x_2$ changes from $\pi$ to 0 and $\phi _3(0)$ changes from 0 to 1, when $\Delta$ changes within a small interval from $- (\sigma ^* - 2)/2$ to 0.

When $x_2<0$ as depicted in Fig. 2, the solution for $\phi _1$ in the range $-2\pi +x_2<x<x_2$ (i.e. for the interface between grain 1 and the liquid) is

12 $$\begin{aligned} \phi _1(x) = \frac{1}{2} \left[ 1 - \cos \frac{x-x_2}{2} \right] = 1-\phi _3(x), \quad x_2<0. \end{aligned}$$

This is the solution for an interface in a two-phases system as for example during dendritic solidification.

### Dynamics of GB premelting

**Figure 3:**
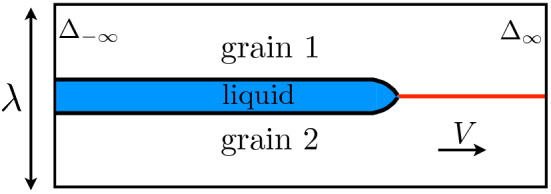
Schematics of the two-dimensional steady-state growth at velocity *V* of the liquid layer along the dry boundary between grain 1 and grain 2. Ahead of the liquid’s tip, the prescribed dimensionless temperature is $\Delta _\infty$, and far behind the tip, the system equilibrates at a dimensionless temperature $\Delta _{-\infty }$.

As mentioned in the Introduction, the main objective of this paper is studying the premelting dynamics. We consider a set up presented in Fig. 3 where the liquid layer propagates at a constant velocity *V* along the dry grain 1/grain 2 boundary in a two-dimensional system with a width $\lambda$. The growth is accompanied by heat diffusion and the system is insulated. Thus no heat flux is assumed at the lower and upper boundaries of the box, i.e. in the direction normal to the growth. The temperature $T_\infty$ of the dry GB is represented by the dimensionless quantity

13 $$\begin{aligned} \Delta _\infty = L \eta (T_\infty - T_m)/(4 \sigma _{sl} T_m), \end{aligned}$$

which is a given of the problem, and which is the temperature that is prescribed at the right boundary of the box. During steady-state growth, as we will see later, heat fluxes are present in a restricted region around the tip of the liquid layer, and, far enough behind, the system equilibrates at a temperature $T_{-\infty }$ represented by

14 $$\begin{aligned} \Delta _{-\infty } = L \eta (T_{-\infty } - T_m)/(4 \sigma _{sl} T_m), \end{aligned}$$

and, in general, $\Delta _{-\infty } \ne \Delta _\infty$. The equilibration far behind the tip leads to the one-dimensional equilibrium described in the previous section, with $\Delta _{-\infty }$ playing the role of $\Delta$.

This set-up is typical for the study of a confined growth regime, for example the dendritic growth in a channel. The new phase, here the liquid, is assumed to nucleate at the GB and subsequently the liquid layer enters the growth regime. This scenario is characteristic of a first-order phase transition for which thermal fluctuations are needed and an energy barrier has to be overcome in order to locally produce a seed of the new phase. While premelting at free surfaces does not seem to require such an energy barrier and takes place homogeneously over the surface^19^, it should be different in the case of a grain boundary since an elastic accommodation is in general required due to the difference in atomic density between the solid and liquid states^36^. We thus assume that nucleation events are rare enough and the nucleii are far enough from each other, so that the liquid layer is able to grow steadily along the GB. Therefore, our set-up corresponds to a situation where the polycrystalline structure, stable at low temperature, is suddenly brought to a higher temperature $\Delta _\infty$. In an experimental situation, a time is needed for the system to reach this temperature; hence, our set-up is relevant to experiments when the time for nucleation is larger than the time for thermal equilibration at $\Delta _\infty$. Note that such a thermal equilibration may take place on a very short time scale in a thin-sample with two-dimensional dynamics as it is the case here.

The description of the pre-melted equilibrium in the previous section allows to find, for a given temperature, the geometrical characteristics of the liquid layer, i.e. $x_1$ and $x_2$. In the case of a growth of the liquid layer at constant velocity *V*, conservation of energy implies that the energy far ahead of the tip equals the energy averaged over the direction perpendicular to growth far behind the tip where equilibration takes place. In other words, the enthalpy associated with the creation of the liquid is compensated by changes in temperature and interface energy. This yields an equation that relates $\Delta _{-\infty }$ to $\Delta _\infty$, through the equilibrium profiles $\phi _i(x)$ given in the previous subsection ($i=1,2,3$) and their spatial derivatives $\phi _i'(x)$:

15 $$\begin{aligned}&\lambda \Delta _{\infty } + \frac{2\pi }{S} \sigma ^* \nonumber \\&\quad =\lambda \Delta _{-\infty } + \frac{1}{S} \int _{-x_1}^{x_1}\left\{ {\tilde{L}} \phi _3^2(3-2\phi _3) - 16\phi _1'\phi _3' + 4\phi _1\phi _3 - 16 \sigma ^* \phi _1'\phi _2' + 4 \sigma ^* \phi _1\phi _2 -16 \phi _2' \phi _3' + 4\phi _2\phi _3\right\} dx \end{aligned}$$

where ${\tilde{L}} = \eta L/\sigma _{sl}$ and $S=4 c_p T_m/L$ with $c_p$ the specific heat. The left hand-side of Eq. (15) corresponds to the energy far ahead of the tip, while the right hand-side corresponds to the energy far behind the tip.

**Figure 4:**
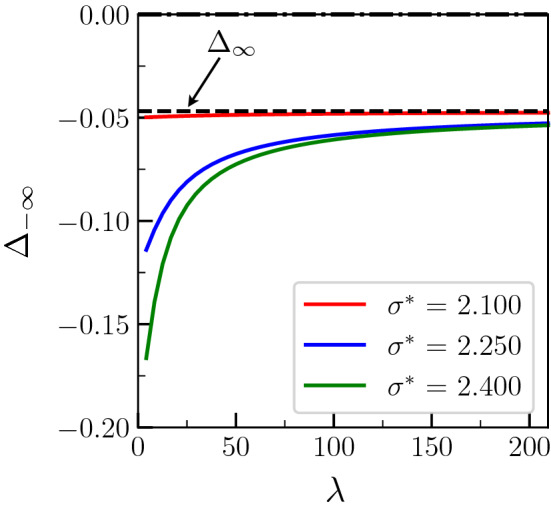
Temperature $\Delta _{-\infty }$ in the equilibrated region far behind the tip as a function of $\lambda$ for different values of $\sigma ^*$ and for a prescribed temperature $\Delta _\infty = -0.047$ of the dry GB (see text for the values of the other parameters). The horizontal dashed line corresponds to $\Delta _\infty$.

**Figure 5:**
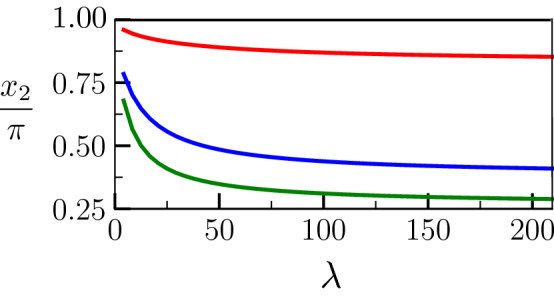
Value of $x_2$ corresponding to Fig. 4.

This equation may be solved numerically by finding which $\Delta _{-\infty }$, associated with its corresponding $x_1$ and $x_2$, is fulfilling the condition (15). It can be seen that the solution for $\Delta _{-\infty }$ depends on the degree of confinement $\lambda$. In Figs. 4 and 5, we present the dependence on $\lambda$ of $\Delta _{-\infty }$ and $x_2$ respectively, for three different values of $\sigma ^*>2$, and for $\Delta _\infty = -0.047$, $S=40$ and ${\tilde{L}} = 15$. These values for *S* and ${\tilde{L}}$, that can correspond for example to $T_m=800$ K, $L=400$ J cm$^{-3}$, $c_p = 5.0$ J cm$^{-3}$ K$^{-1}$, $\sigma _{sl} =$0.2 J m$^{-2}$, and a characteristic width of the liquid layer $\eta = 7.5$ nm, are typical for metals. In particular, they are close to the properties of pure Al, for which some experimental results are compared to our polycrystalline simulation later on. In Figs. 4 and 5, it can be seen that $\Delta _{-\infty }$ increases with $\lambda$ and $x_2$ decreases when $\lambda$ increases, corresponding to an increases in the width of the liquid layer when $\lambda$ increases. At large $\lambda$, $\Delta _{-\infty }$ converges for all $\sigma ^*$ to the dashed line that corresponds to $\Delta _\infty$. The limit $\Delta _{-\infty } (\lambda \rightarrow \infty ) \rightarrow \Delta _\infty$ results from the fact that the terms proportional to $\lambda$ then dominate in Eq. (15), the energy increase caused by the presence of the liquid layer (responsible for $\Delta _{-\infty } \le \Delta _\infty$) becoming negligible in the energy conservation relation. Correspondingly, the geometry of the liquid layer represented by $x_2$ in Fig. 5 becomes independent of $\lambda$ when $\lambda \gg 1$, and may be estimated replacing $\Delta$ by $\Delta _\infty$ in Eqs. (10) and (11).

On the other hand, when $\lambda$ becomes small, it can be seen from Fig. 5 that $x_2$ goes to $\pi$. As mentioned in the previous paragraph, this situation corresponds to the onset of premelting, and indeed $\Delta _{-\infty } (\lambda \rightarrow 0) \rightarrow - (\sigma ^*-2)/2$ in Fig. 4. Let us note however that $\lambda$ may not be arbitrarily small since it should, at least, exceed the interface width, i.e. 2$\pi$ in our dimensionless units.

**Figure 6:**
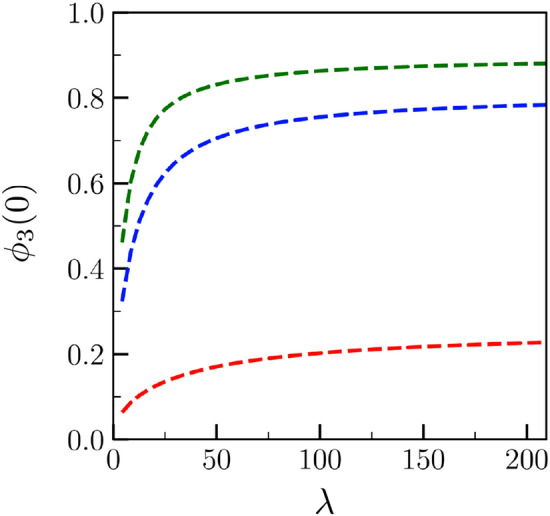
Level of disorder of the growing liquid layer $\phi _3(0)$ [see Eq. (9)] corresponding to Fig. 4.

The $\lambda$-dependence of $\Delta _{-\infty }$ and $x_2$ implies a $\lambda$-dependence of the level of disorder of the liquid layer that grows along the dry GB. As can be indeed seen in Fig. 6, while the level of disorder $\phi _3(0)$ given in Eq. (9) intuitively increases with $\sigma ^*$, it also decreases when $\lambda$ decreases (since $\Delta _{-\infty }$ increases with $\lambda$).

Let us note that, as a consequence of the fact that $\Delta _\infty$ is close to $- (\sigma ^*-2)/2$ for $\sigma ^* = 2.1$, the variations of $\Delta _{-\infty }$ and $x_2$ with $\lambda$ are small. Thus $\Delta _{-\infty }(\lambda ) \rightarrow - (\sigma ^*-2)/2$ and $x_2(\lambda ) \rightarrow \pi$ when $\Delta _\infty \rightarrow - (\sigma ^*-2)/2$. Therefore, a critical undercooling of the dry GB may be defined as

16 $$\begin{aligned} \Delta _\infty ^{(1)} = - (\sigma ^*-2)/2 \end{aligned}$$

describing the fact that, even if $\sigma ^*>2$, the growth of the liquid layer along the dry GB does not occur when $\Delta _\infty <\Delta _\infty ^{(1)}$.

Let us now consider the case corresponding to the other transition, i.e. the onset of melting for which $x_2=0$ and $\Delta =0$ in Eqs. (10) and (11). Setting $\Delta _{-\infty }=0, x_2=0$ and $x_1=2\pi$ in Eq. (15) and in the equilibrium profiles, we find a new critical value for $\Delta _\infty$:

17 $$\begin{aligned} \Delta _\infty ^{(2)} = \frac{2 \pi }{ S }\; \frac{ {\tilde{L}} - (\sigma ^* - 2) }{\lambda }. \end{aligned}$$

When $\Delta _\infty < \Delta _\infty ^{(2)}$, premelting occurs with $\Delta _{-\infty }<0$, $x_2>0$ and $\phi _3(0)<1$. Conversely, when $\Delta _\infty > \Delta _\infty ^{(2)}$, melting occurs with $\Delta _{-\infty } = 0$, $x_2<0$ and $\phi _3(0) = 1$, as depicted in Fig. 2. For the typical values used previously and yielding ${\tilde{L}}=15$, it can be seen that $\Delta _\infty ^{(2)}>0$. Thus, from our theoretical analysis of energy conservation during steady-state growth of the liquid layer, we find that premelting may take place even when the temperature to which the dry GB is brought lies above the melting temperature. We are not aware of any analysis in the literature pointing in that direction, probably because the dynamics in such a situation has rather scarcely been studied. In Refs.^35,37^, steady-state melting along a dry GB was investigated when $\sigma ^*-2<0$ in an infinite system ($\lambda \rightarrow \infty$). Let us note that the steady-state velocity then diverges when $\sigma ^*-2$ approaches 0.

The $\lambda$-dependence of the critical temperature $\Delta _\infty ^{(2)}$ implies that, for a given $\Delta _\infty$, the transition from premelting to melting may occur at $\lambda =\lambda _c= 2\pi [{\tilde{L}} - (\sigma ^*-2)]/(\Delta _\infty S)$ when varying $\lambda$.

**Figure 7:**
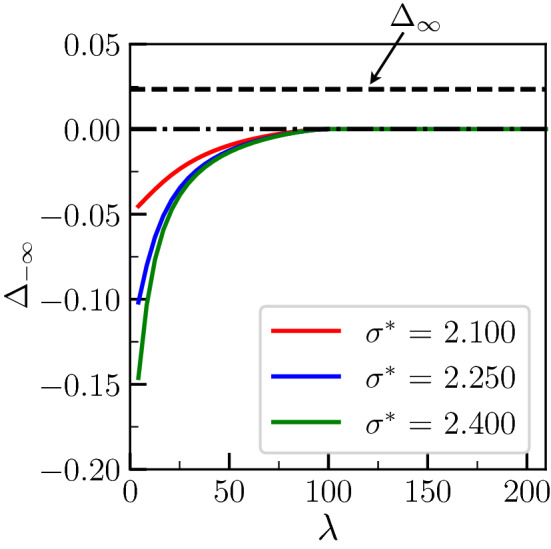
Temperature $\Delta _{-\infty }$ in the equilibrated region far behind the tip as a function of $\lambda$ for different values of $\sigma ^*$ and for a prescribed temperature $\Delta _\infty = 0.023$ of the dry GB (see text for the values of the other parameters). The horizontal dashed line corresponds to $\Delta _\infty$. For each $\sigma ^*$, there exists a $\lambda _c$ such that for $\lambda >\lambda _c$, the transition corresponds to melting with $\Delta _{-\infty }=0$.

**Figure 8:**
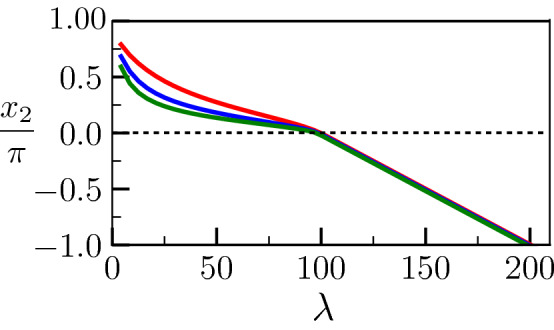
Value of $x_2$ corresponding to Fig. 7. The variation is linear as soon as the melting regime is reached.

**Figure 9:**
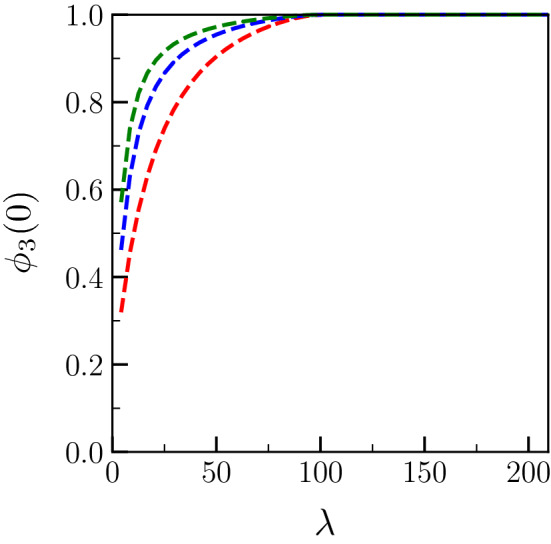
Level of disorder of the growing liquid layer $\phi _3(0)$ [see Eq. (9)] corresponding to Fig. 7. The degree of disorder is characteristic of the bulk liquid ($\phi _3=1$) as soon as the melting regime is reached.

This is what is shown in Figs. 7, 8, and 9, where we plot respectively $\Delta _{-\infty }, x_2/\pi$ and the degree of disorder of the liquid layer $\phi _3(0)$ as a function of $\lambda$ for a prescribed overheating $\Delta _\infty = 0.023$, and for the same parameters as for Figs. 4, 5, and 6.

In Fig. 7, it can be seen that, in contrast to Fig. 4, $\Delta _{-\infty }$ does not converge to $\Delta _\infty$ when $\lambda \rightarrow \infty$. Instead, $\Delta _{-\infty }$ reaches 0 at $\lambda = \lambda _c$, and remains equal to 0 for larger $\lambda$. In Fig. 8, it can be seen that $x_2$ decreases from $\pi$ to 0 when $\lambda$, smaller than the critical value, increases. Then, for $\lambda >\lambda _c$, $x_2$ follows $x_2 = - (\lambda -\lambda _c) \Delta _\infty S/(2{\tilde{L}})$. In Fig.9, while $\phi _3(0) <1$ when $\lambda <\lambda _c$, $\phi _3(0)=1$ when $\lambda >\lambda _c$. Here, in these plots, the fact that ${\tilde{L}}$ is much larger than $\sigma ^*-2$ explains why $\lambda _c$ is very similar for all three curves.

**Figure 10:**
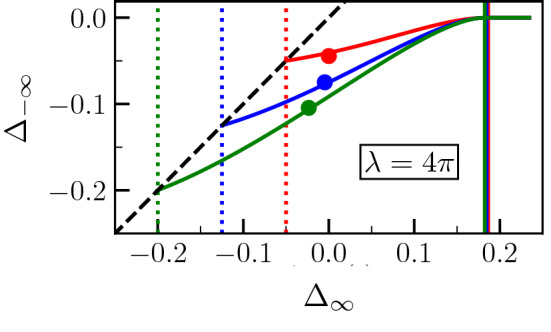
For a given $\lambda =4\pi$, plot of the dimensionless temperature in the equilibrated region far behind the tip $\Delta _{-\infty }$ as a function of the dimensionless temperature ahead of the tip $\Delta _\infty$ for $\sigma ^* = 2.1, 2.25, 2.4$ (same color coding as in the previous figures). The vertical dotted lines correspond to $\Delta _\infty ^{(1)}$, and the vertical solid lines correspond to $\Delta _\infty ^{(2)}$. The dots correspond to phase field simulations, demonstrating an accurate conservation of energy.

If now we fix $\lambda$, we may present $\Delta _{-\infty }$ as a function of $\Delta _\infty$. This is what is displayed in Fig. 10 for $\lambda =4\pi$, and for the three values of $\sigma ^*$ that are used in the previous figures. The vertical dotted lines correspond to $\Delta _\infty ^{(1)}$ and for $\Delta _\infty <\Delta _\infty ^{(1)}$ no premelting exists. The vertical solid lines correspond to $\Delta _\infty ^{(2)}$ and for $\Delta _\infty >\Delta _\infty ^{(2)}$ melting takes place with $\Delta _{-\infty }=0$. The oblique dashed line represents $\Delta _{-\infty } = \Delta _\infty$, i.e. the limiting case for $\lambda \rightarrow \infty$, for which $\Delta _\infty ^{(2)} \rightarrow 0$. The dots correspond to phase-field simulations.

**Figure 11:**
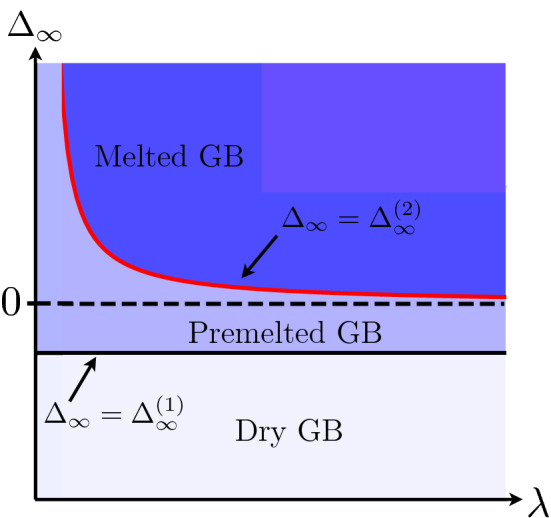
Kinetic phase diagram where we give, as a function of $\Delta _\infty$ and $\lambda$, the nature of the transformation: the GB remains dry when $\Delta _\infty < \Delta _\infty ^{(1)}$, the dry GB transforms into a pre-melted GB with an atomically thin liquid layer for $\Delta _\infty ^{(1)}< \Delta _\infty < \Delta _\infty ^{(2)}$, and the dry GB transforms into a macroscopic liquid–solid equilibrium with a liquid layer having a macroscopic width when $\Delta _\infty ^{(2)} < \Delta _\infty$.

To summarize the phenomenology associated with the two critical values of $\Delta _\infty$, we present in Fig. 11 a schematic kinetic phase diagram where, as a function of $\Delta _\infty$ and $\lambda$, the equilibrium state after transformation is given. For $\Delta _\infty < \Delta _\infty ^{(1)}$, no transformation occurs. For $\Delta _\infty ^{(1)}<\Delta _\infty < \Delta _\infty ^{(2)}$, the dry GB transforms into a pre-melted GB with $x_2>0$ and for $\Delta _\infty >\Delta _\infty ^{(2)}$ melting occurs with $x_2<0$. The interval of temperature for which the dry GB transforms into a pre-melted GB increases when $\lambda$ decreases.

Let us note that, in the case where the disjoining potential’s dependence on the layer’s width is non-monotonous, for example yielding a thin-to-thick transition mentioned in the Introduction, corresponding complications of the scenarios presented above for $\Delta _{-\infty }, x_2$ and $\phi _3$ occur and turning points appear. In this case, the sign of the disjoining pressure, i.e. the derivative of the disjoining potential with respect to the layer’s width, alternates, and $\Delta _{-\infty }$ is positive when the layer’s width corresponds to an attractive part of the disjoining potential. We have nevertheless checked using a sharp-interface analytical approach using the disjoining potential proposed in Ref.^25^ that $\Delta _{-\infty }<\Delta _\infty$ in any case.

To conclude before presenting the simulations, the phase field model allows for a unified description including the microscopic physics at the atomic scale (represented by the scale of the interface width in the phase field model) responsible for premelting, and reproduces the macroscopic limit, for which the atomic distance formally vanishes, with the width of the liquid layer growing proportionally to $\lambda$ when $\lambda >\lambda _c$. Conversely, when $\lambda$ is fixed, the width of the liquid layer decreases when $\Delta _\infty$, larger than $\Delta _\infty ^{(2)}$, decreases, and it reaches the atomic scale at $\Delta _\infty \sim \Delta _\infty ^{(2)}$. As schematically represented in Fig. 11, the smaller $\lambda$, the larger $\Delta _\infty$ should be in order for the liquid layer to achieve a macroscopic width. The difference between $\Delta _\infty$ and $\Delta _{-\infty }$ (which exactly equals $\Delta _\infty ^{(2)}$ when $\Delta _\infty = \Delta _\infty ^{(2)}$ since $\Delta _{-\infty } = 0$ in this case) increases when $\lambda$ decreases (with roughly a $1/\lambda$ dependence). For the example displayed in Fig. 10 with $\lambda =4\pi$, those differences are of order 0.1, corresponding to variations of temperature of order 20 K. These huge temperature differences are reached because $\lambda$ is extremely small (approximately few tens of atoms). If we identify $\lambda$ with the grain size of a polycrystalline structure, such a small $\lambda$ is obviously unrealistic even for structures that are produced at extremely large cooling rates. For grains about few $\upmu$m in size, typically seen in laser-based additive manufacturing, we expect the temperature differences to be of the order of 0.1 K.

## Phase field simulations

**Figure 12:**
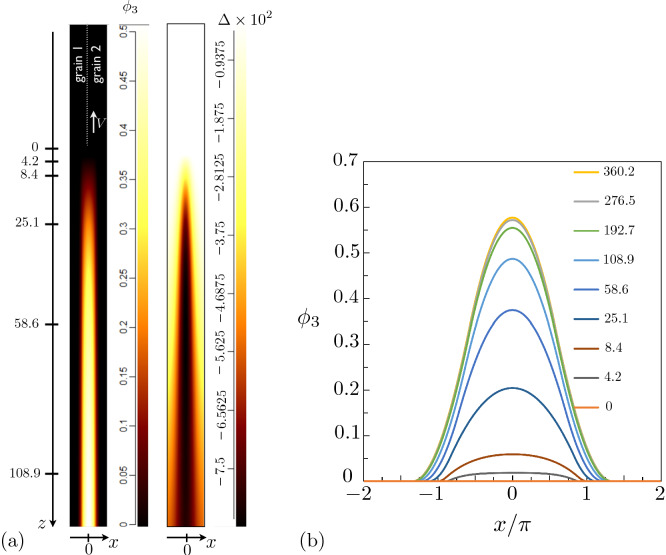
(**a**) Part of the simulations box, with a color map for $\phi _3(x,z)$ (left) and the dimensionless temperature $\Delta (x,z)$ (right). The dry GB at $z \rightarrow -\infty$ is represented by the vertical white dotted line, and the growth takes place in the direction of the steady-state velocity *V*. At the formal position of the triple junction $(x=0,z=z_0)$, we have $\phi _1=\phi _2=\phi _3=1/3$. At larger *z*, the liquid layer approaches the equilibrium given by Eq. (15). The temperature far ahead of the liquid layer’s tip ($z \rightarrow -\infty$) is $\Delta _\infty = -0.0046875$; (**b**) $\phi _3$ profiles in the *x*-direction at different *z*-positions, displayed in (**a**). The equilibration occurs at $z \simeq 300$ as illustrated by the small difference between $\phi _3$ at *z*=276.5 and *z*=360.2.

**Figure 13:**
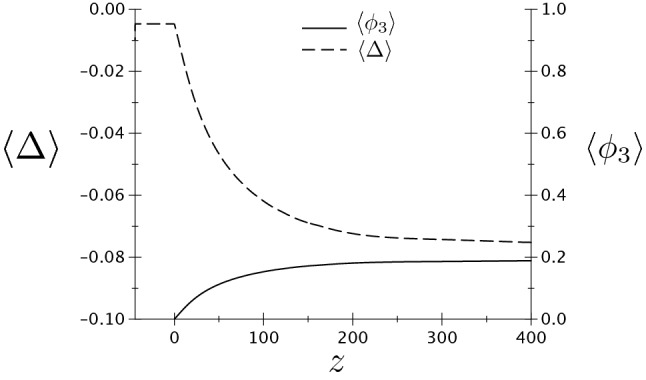
Dependence in the *z*-direction of the phase field $\phi _3$ and the dimensionless temperature $\Delta$ averaged over the *x*-direction (see text) for the simulation presented in Fig. 12.

In this section, simulations of the growth dynamics of the liquid layer along a dry GB are presented. In order to obtain the correct conservation of energy in the simulations, we set the grid spacing to 1/75 of the interface width $\eta$ and simulated domains that are very elongated in the growth direction *z*. From Fig. 10, it can be seen that the temperature of the equilibrated region far behind the tip obtained from the simulations (the dots) agrees well with the one calculated analytically (the solid lines). The diffusion coefficient is set constant throughout the whole simulation domain, i.e. the diffusivity is equal in the liquid and solid phase. In Fig. 12a, where actually only a portion of the simulation is displayed and where the *z* axis is such that the steady-state velocity is negative, we present the map for the degree of disorder $\phi _3(x,z)$ and for the dimensionless temperature field $\Delta (x,z)=L\eta [T(x,z)-T_m]/(4\sigma _{sl} T_m)$ (for a simulation at $\Delta _\infty = -0.0046875$, $\sigma ^*=2.25$, $\lambda =4\pi$ and otherwise the same parameters as for Fig. 10, on which we denote $\Delta _{-\infty }$ obtained from the simulation using dots). In the region $z<0$, the GB is dry with $\phi _3=0$, and $\Delta = \Delta _\infty$. At small positive *z*, we distinguish a diffuse region where $\phi _3$ emerges, and, as *z* increases the liquid layer achieves progressively its equilibrium structure. In Fig. 12b, the profiles of $\phi _3$ along the x direction *x*, which is perpendicular to growth direction *z*, are displayed for *z* values exhibited in Fig. 12a. It can be seen that only at position $z \approx 300$, the liquid layer reaches its equilibrium structure, with the $\phi _3$ profile changing very slightly between $z=276.5$ and $z=360.2$. This is the distance to the tip of the liquid layer needed for the temperature field to equilibrate. It is obvious that simulating such a process using an atomistic method such as phase-field-crystal or molecular dynamics would be computationally intractable. In Fig. 13, the *z*-dependence of the phase field $\phi _3$ and the dimensionless temperature $\Delta$ averaged over the *x*-direction, i.e. $\langle \phi _3 \rangle (z) = \int _x (dx/\lambda ) \phi _3 (x,z)$ and $\langle \Delta \rangle (z) = \int _x (dx/\lambda ) \Delta (x,z)$, are presented. It can be seen that $\langle \Delta \rangle$ starts to deviate from $\Delta _\infty$ and heat fluxes establish as soon as $\langle \phi _3 \rangle$ deviates from 0. The latter deviation is linear, illustrating the fact that, as can be seen in Fig. 12b, the bump that characterizes $\phi _3$ in the neighborhood of $z=0$ has a width that varies weakly with *z*, while its amplitude varies much stronger. The saturation of $\langle \phi _3 \rangle$ and $\langle \Delta \rangle$ at large *z* indicates that, far behind the tip, heat fluxes vanish and the system equilibrates.

### Structure of the triple junction

**Figure 14:**
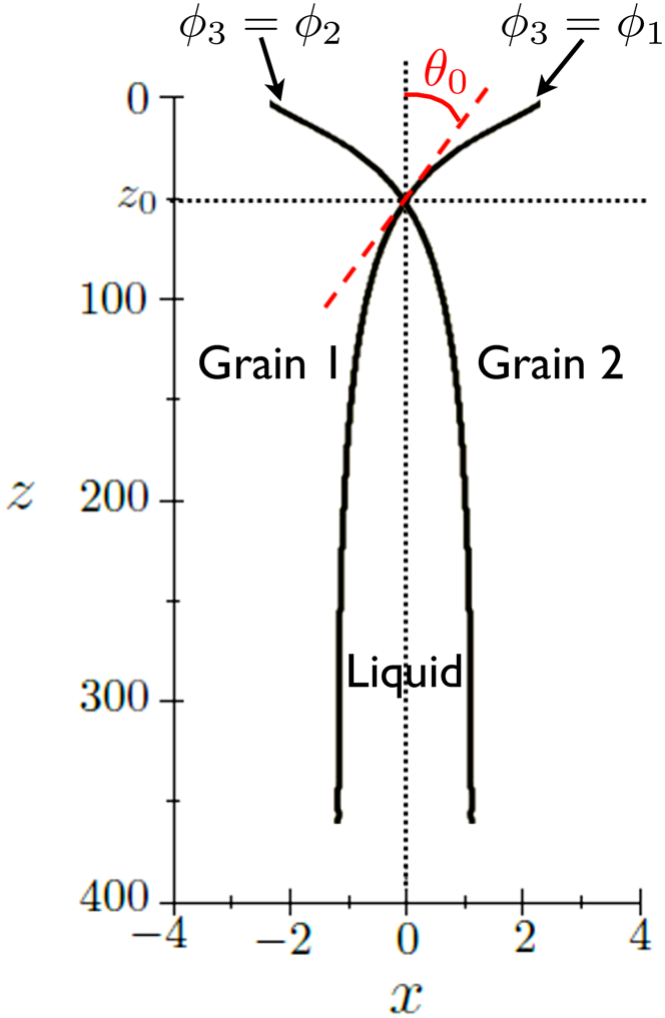
Isolines $\phi _1=\phi _3$ and $\phi _2=\phi _3$ in the neighborhood of the triple junction, located at ($x=0, z=z_0$) and corresponding to $\phi _1=\phi _2=\phi _3=1/3$. The contact angle $\theta _0$, given by the slope of the isolines at $z_0$, is close to 2$^\circ$.

An important feature of the phase field results concerns the structure close to the tip of the liquid layer. Here, we focus only on the simulation corresponding to Fig. 12. In the macroscopic approach, an equilibrium in the form of Young’s law at the triple junction between grain 1, grain 2 and the liquid layer is not possible for $\sigma ^*-2>0$, and the liquid should wet the GB with a vanishing macroscopic contact angle. Obviously in our case this picture is not appropriate since we study the propagation of the transition region that connects spatially the dry GB to the wet GB. We therefore focus on the behavior of the phase fields, shown for $\phi _3$ in Fig. 12, on the scale of the interface width. We explore the structure close to the tip in a way similar to what is presented in Fig. 9 in Ref.^38^. We locate the two isolines $\phi _1=\phi _3$ and $\phi _2=\phi _3$, and the intersection of these two isolines, at $x=0, z=z_0$, yields the position of the triple junction where $\phi _1=\phi _2=\phi _3=1/3$. The microscopic contact angle $\theta _0$ is then defined by the angle between the isolines and the vertical axis at the triple junction. We illustrate this procedure in Fig. 14 and find $\theta _0 \simeq 2^\circ$.

In Ref.^35^, a generalization of Young’s law for a triple junction at melting temperature is proposed in order to include effects of structural forces in the case $\sigma ^*<2$. The microscopic contact angle $\theta _0$ relates the macroscopic contact angle $\theta _\infty$ and $\sigma ^*$ through $\theta _\infty ^2 - \theta _0^2 = -(\sigma ^*-2)$. In the case $\sigma ^*>2$, their formula may actually be read by setting $\theta _\infty = 0$ (according to classical Young’s law), and one finds a finite microscopic contact angle $\theta _0 = \sqrt{\sigma ^*-2}$ and a well-defined triple junction. We see that our simulation does not reproduce this result with a $\theta _0$ much smaller than $\sqrt{\sigma ^*-2}$. Several arguments may be invoked in order to explain this discrepancy. First, the analysis in Ref.^35^ holds for a triple junction at melting temperature, while, in our simulation, the temperature field is highly inhomogeneous on the scale of the interface width and the temperature lies well below $T_m$ where the triple junction is defined. Second, their analysis is performed within a small slope approximation that requires $\sigma ^*-2 \ll 1$, which is not the case in our simulation. Finally, as mentioned in Ref.^35^, kinetic effects at the triple junction may take place yielding a deviation from Young’s law for the macroscopic contact angle. All these effects are interesting but their investigation is beyond the scope of this paper.

We note also that the procedure outlined above and illustrated in Fig. 14 is enabled by the fact that $\phi _3>1/3$ in the equilibrated region far behind the tip, as can be seen in Fig. 12. Thus, it is clear that when $\phi _3$ remains smaller than 1/3, as for example for small enough $\lambda$, such a procedure may not be adopted. This case lies also beyond the scope of this paper.

### Velocity dependence on surface energies

**Figure 15:**
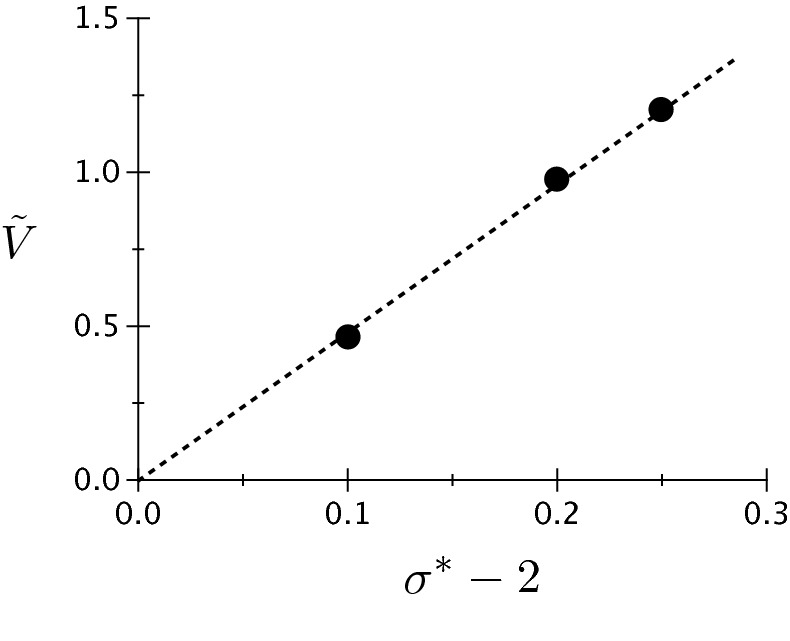
Dimensionless steady-state velocity (see text for definition) as a function of the normalized interface energy difference characteristic of premelting $\sigma ^*-2 = \sigma _{gb}/\sigma _{sl} -2$ for a dry GB at melting temperature, i.e. $\Delta _\infty =0$.

**Figure 16:**
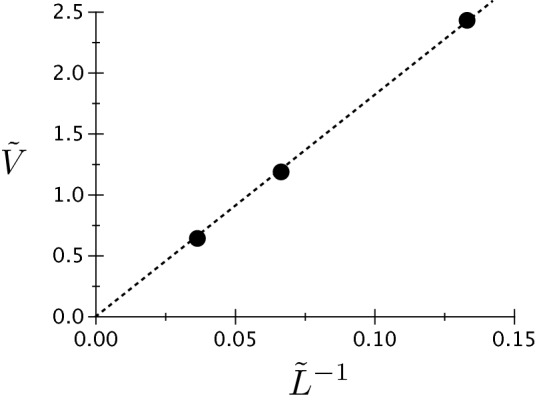
Dimensionless steady-state velocity (see text for definition) as a function of the dimensionless solid–liquid interface energy ${\tilde{L}}^{-1}= \sigma _{sl}/(L \eta )$ for $\Delta _\infty = -0.0046875$.

Let us now focus on the dependence of the tip growth velocity on the surface energies. For this purpose, we define the dimensionless velocity

18 $$\begin{aligned} {\tilde{V}} = \frac{1}{S} \frac{V \eta }{D} \end{aligned}$$

where *V* is the steady-state velocity and *D* is the heat diffusion coefficient. The normalization of the dimensionless velocity by 1/*S* comes from the following arguments. Our definition of $\Delta$ in Eq. (8) differs from the usual definition of the dimensionless driving force for heat diffusion controlled processes in sharp interface models $\Delta _{\text {sh}} = c_p(T-T_m)/L = S \Delta / {\tilde{L}}$. Since the growth velocity typically scales with a positive power of $\Delta _{\text {sh}}$, it vanishes when $c_p/L \rightarrow 0$, this fact being the reason why we divide our velocity by *S*. As we will see in the following, the definition of ${\tilde{V}}$ given in Eq. (18) provides values of order unity. We set the channel width to $\lambda =4\pi$, and we perform three simulations for each dependence, i.e. on $\sigma ^*$ and on the dimensionless solid–liquid interface energy ${\tilde{L}}^{-1} = \sigma _{sl}/(L\eta )$. In Fig. 15, we plot the growth velocity as a function of $\sigma ^*-2=0.1, 0.2$ and 0.25 for ${\tilde{L}}=15$ and $\Delta _\infty = 0$ (we recall that, in a channel, $\Delta _\infty =0$ does not represent a special point as illustrated by Fig. 10 for example). In Fig. 16, we plot velocity as a function of $\tilde{L}^{-1}=11/300, 1/15$ and 2/15 for $\sigma ^*=2.25$ and $\Delta _\infty = -0.0046875$. We see that for both curves, the data is well fitted using a straight line passing through the origin. This suggests that $V \propto (\sigma ^*-2)\sigma _{sl}=\sigma _{gb}-2\sigma _{sl}$. Thus, it seems that in the premelting problem, the excess of interface energy attributable to the unfavorable dryness of the GB is the driving force. This kind of dependence of steady velocity on the interface energy is rather unusual for a growth process in a solid–liquid system. Indeed, in dendritic growth for example, the velocity is inversely proportional to the solid–liquid energy, the latter hindering the growth owing to the energetic penalization of interface curvature. Closer to our current interest, the steady-state velocity scales with the inverse of the solid–liquid interface energy also in the theories developed in Refs.^35,37^ for melting along a GB. In the second part of the following discussion, we comment on these theories and on their link with our premelting problem.

Let us comment on the values of the steady-state velocity that we obtain in phase field simulations. Using a typical value $D=10^6$ m$^2$/s for the heat diffusivity and the values for $\eta$ and *S* used previously, i.e. $\eta = 7.5$ nm and $S=4c_P T_m/L = 40$, we find velocities of order 10$^3$ m s$^{-1}$. Such a value is questionable because it is in the order of the speed of sound. In our simulations, we have used a mobility $M_{ij}$ (see “Methods”) providing equilibrium boundary conditions at the interface within the thin interface limit of the phase-field model^39^. Such a choice corresponds to an infinite interface mobility within the sharp interface limit for conditions close to equilibrium. Then, for sufficiently small velocities, the interface mobility does not play any role, neither in the pattern nor in the velocity selection mechanisms. Here, we have found that reducing the phase field mobility $M_{ij}$ does influence the growth. More precisely, reducing the phase field mobility $M_{ij}$ by a factor 30, approximately decreases the steady-state velocity by the same factor 30. This indicates that the kinetic does not follow the diffusion-controlled regime for which interface mobility does not play a role. On the other hand, for a purely kinetically-controlled regime, one should obtain a homogeneous temperature field, which here is not the case either (see Fig. 12). Thus, it is likely that the growth kinetics of pre-melted GBs falls into a mixed-mode regime where the interface mobility is a material related quantity that influences the transformation speed. This is known for other fast transformations, such as rapid crystallization in phase change materials^40^. The results may also be applied to premelting in an alloy. In this case, the diffusion coefficient in the liquid layer is probably in the order of a bulk liquid diffusion coefficient, i.e. typically three orders of magnitude smaller than the heat diffusivity provided above, and the diffusion coefficient in the solid grains is typically six orders of magnitude smaller than this heat diffusivity. If one assumes that the diffusion in the solid grains mainly sets the magnitude of the liquid layer growth velocity, the latter then becomes of the order of a mm/s, which is now well below the speed of sound. Let us note moreover that when one applies the classical dendritic growth theory to the melting of an alloy, for which the diffusion coefficient is much larger in the growing phase than in the disappearing phase, the velocity does not scale as $D_S$ but as $D_S^2/D_L$ where $D_S$ ($D_L$) is the diffusion coefficient in the solid (liquid)^41^. This suggests that premelting in an alloy might even take place at velocities smaller than a mm/s.

## Simulation of a polycrystalline evolution

**Figure 17:**
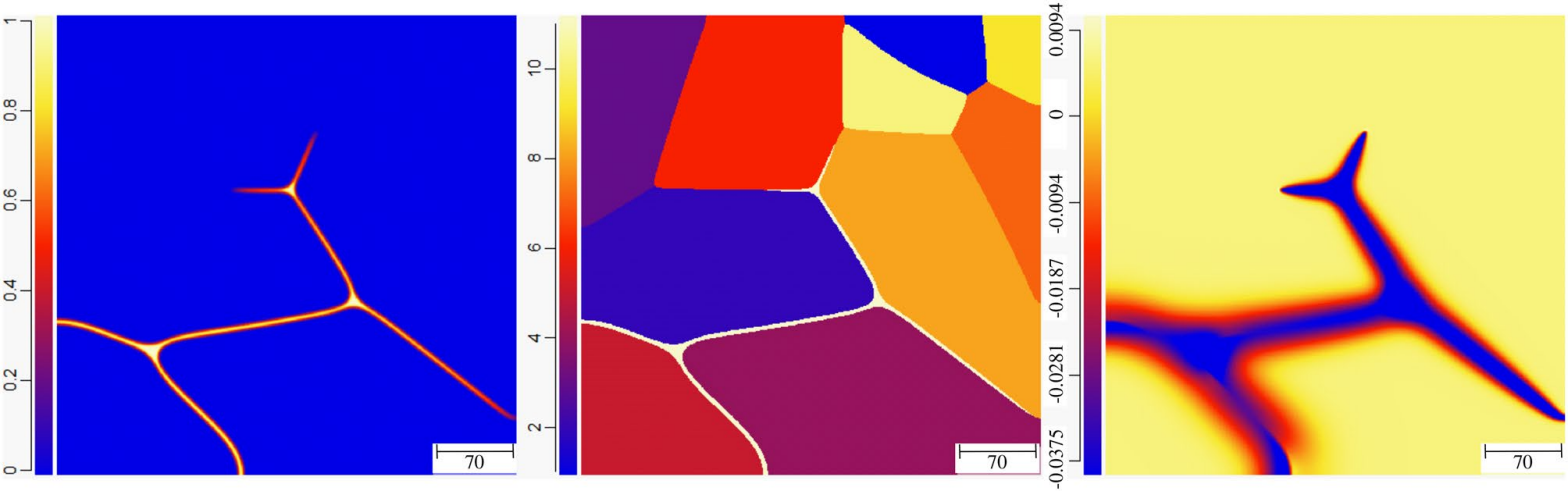
Color map for $\phi _3$ (left), grain number (middle), and dimensionaless temperature $\Delta$ (right) for a polycrystalline structure initially at $\Delta = 0.0046875$, at some intermediate time during the propogation of the pre-melted layer along the dry GBs.

Now, we briefly investigate the effect of premelting on the evolution of a polycrystalline structure relevant to experiments. We simulated the evolution of a microstructure that, initially, consisted of ten grains and a small liquid domain that enables the premelting liquid layer to propagate along the dry GBs. The GB energies are all equal with $\sigma ^* = 2.25$, and as previously, the system is insulated. The evolution of the system as the liquid pre-melts the dry GBs and as the pre-melted GBs evolve with time can be seen in the “Supplementary Material”. Here, we show only the results at three different points in time. In Fig. 17, an intermediate state for which the liquid layer has not yet propagated along every dry GB is displayed. In the left panel, the color codes $\phi _3$, in the middle panel a color is assigned to each grain, and in the right panel the color codes the dimensionless temperature $\Delta$. We see that the temperature has fallen below $T_m$ in the neighborhood of the liquid layer, while it is still at the initial temperature $\Delta = 0.0046875$ above $T_m$ in the bulk of the grains.

**Figure 18:**
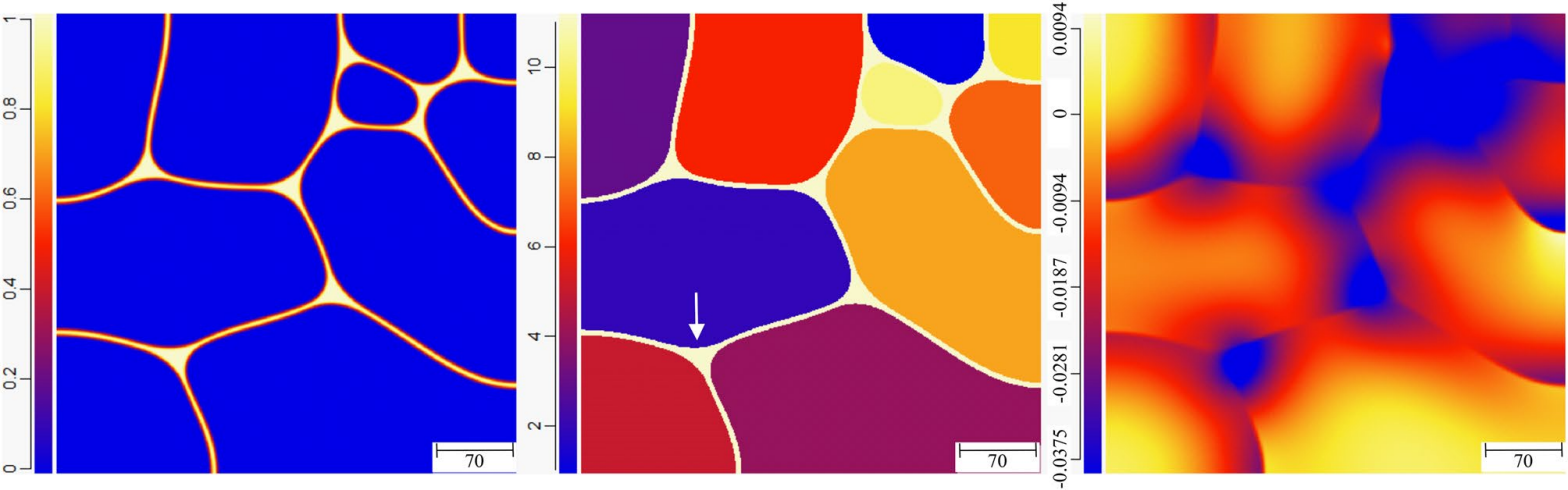
The status at some later time after all GBs have been pre-melted. Liquid pockets of macroscopic size ($\phi$=1) have emerged at the triple junctions, and protrusions, such as the one highlighted by the vertical arrow in the middle panel, allow minimization of the disjoining potential in the adjacent pre-melted liquid layers.

In Fig. 18, the liquid layer has propagated along all GBs; there has been an evolution of the size and shape of the grains; and we observe the heat diffusion field from the center of the grains to the pre-melted GBs. At the triple junctions, relatively large liquid pockets have emerged, where $\phi _3=1$ since the distance between solid/liquid interfaces is much larger than the interface width. Nevertheless, the curvature of the solid/liquid interfaces allows a temperature below $T_m$ in these regions. Interestingly, the protrusion that is highlighted with an arrow in the center panel is already observable at a much earlier stage of the evolution in Fig. 17. This is counterintuitive if, naively, one expects that the protrusion simply enhances the interface energy of the system. Instead, one should note that the curvature of the solid/liquid interface allows a minimization of the disjoining potential in the thin liquid layers adjacent to the triple junction, and is thus energetically favorable. This is an effect of the disjoining potential that should be of fundamental importance for the grain evolution in systems that are prone to premelting, and this point is dicussed further next.

**Figure 19:**
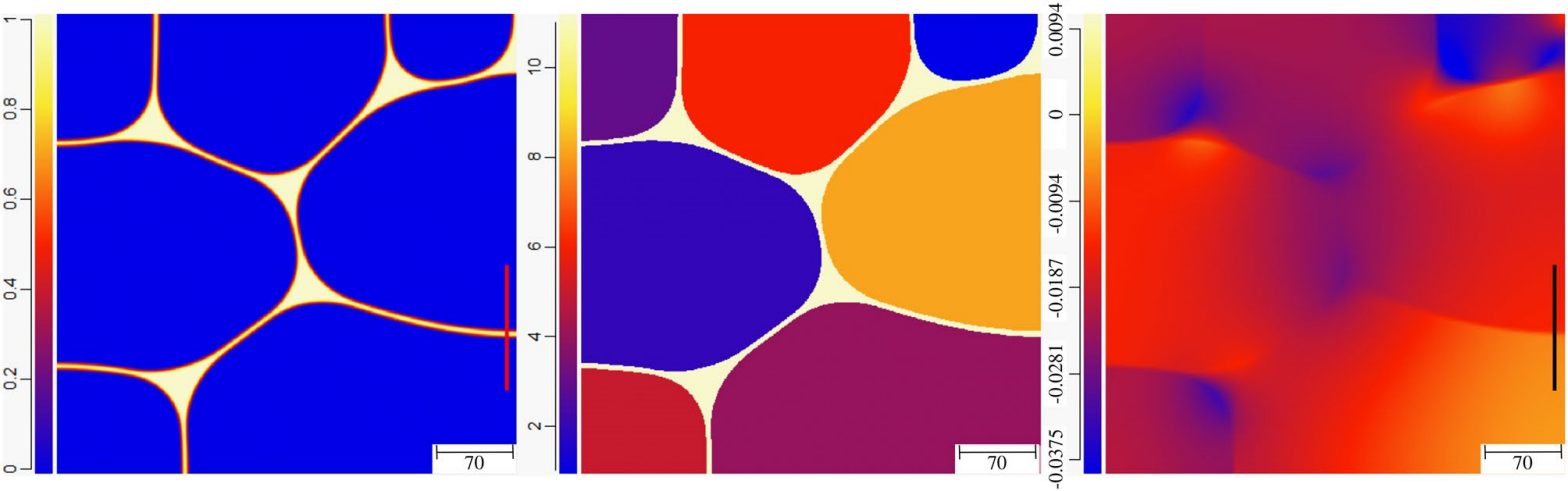
The status at an even later time when grain morphology has evolved further, the temeprature in the whole simulation domain has fallen below $T_m$, and the size of the liquid pockets at the triple junctions has further increased.

As a result of the promotion of premelting against melting due to confinement (here provided by small grain sizes) described in the previous sections, we see in Fig. 19 that the temperature everywhere in the domain is below $T_m$ although $\Delta = 0.0046875$ initially, in accordance with our analysis of steady-state growth of a liquid layer and the associated energy conservation (i.e., Eq. 15). At the stage of the evolution presented in Fig. 19, some grains have increased in size, some have decreased in size, and three grains have vanished. The liquid pockets at the triple junctions have further increased in size, but we still identify the effect of the disjoining potential minimization through unusual shapes of the grains and curvatures of the solid/liquid interfaces. Let us note that such pre-melted polycrystalline structure was nicely evidenced experimentally (see Figs. 3 and 4 in Ref.^42^). These experiments were conducted in pure Al (that has, as mentioned earlier, properties close to the ones that we use here), and the undercooling $\Delta \simeq -0.01$ in our simulation, that corresponds to $(T-T_m)/T_m = 0.01 \times 4/{\tilde{L}} \simeq 0.0027$, is in qualitative agreement with the observation of the authors in^42^ that “no signs of melting were detected for temperatures up to 0.999 $T_m$.

**Figure 20:**
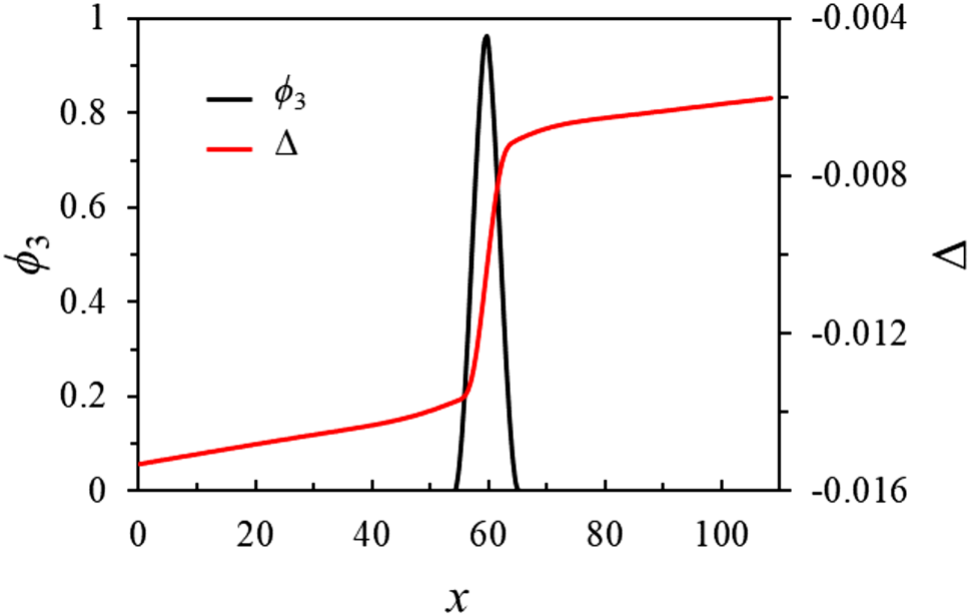
Profiles of $\phi _3$ and $\Delta$ along the vertical line depicted in Fig. 19.

In addition, we have plotted in Fig. 20 the $\phi _3$ and $\Delta$ profiles along the vertical line visible in the left and right panels of Fig. 19. We see that the temperature gradient is much larger across the liquid layer than in the solid. This gradient of temperature across the liquid layer, mainly resulting from the opposite sign of the Gibbs–Thomson effect at the two solid/liquid interfaces, is responsible for the motion of the liquid layer in its normal direction, with a velocity that is easily estimated using the Stefan condition at the interfaces.

**Figure 21:**
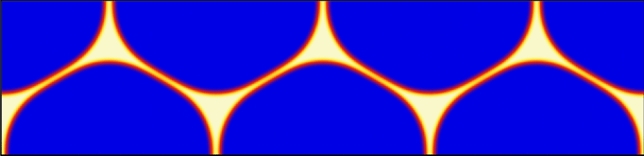
Color map of $\phi _3$ showing the polycrystalline pre-melted equilibrium at a temperature blow $T_m$ with six equivalent grains (all dry GB energies are the same) arranged with hexagonal symmetry. Boundary conditions for $\phi _3$ are periodic at the left and right boundaries and of von neumann type at the top and bottom boundaries.

Let us now present the physical picture that underpins the phenomenology of the polycrystalline evolution discussed above. First, note that a polycrystalline equilibrium state exists for $\Delta < 0$, i.e. at a temperature below $T_m$, as shown in Fig. 21 (see the caption for more details). As already mentioned, when the size of the liquid pocket at the triple junctions is large enough, the liquid possesses the characteristics of the bulk liquid ($\phi _3=1$) at least at its center.

Of course, by symmetry, if the dry GB energies are equal, the angles between the pre-melted GBs will be the same (i.e., $2 \pi /3$); if those energies are not equal, the angles will still be the same if the radius of the curvature of the solid/liquid interfaces bounding the liquid pocket is much larger than the width of the layer, i.e., when $|\Delta | \ll 1$. Then, in an out-of-equilibrium scenario similar to the one discussed in Fig. 19, if grains are large enough, the curvature of the pre-melted layer will be small enough to make the temperature variations across the liquid layer much smaller than $|\Delta |$. In that case, the three lenght scales (the width of the pre-melted liquid layers $\eta$, their radius of curvature *R* of the order of grain size, and the radius of curvature of the liquid pockets $\eta /|\Delta |$) will be fully separated; the triple junction will be close to the equilibrium presented in Fig. 21; and the normal velocity of the pre-melted liquid layers will be proportional to their curvature, corresponding precisely to a curvature-driven phenomenon as for the commonly accepted dynamics of dry GBs. However, here, the mobility of pre-melted GBs, i.e. the proportionality coefficient between velocity and curvature, will be given by the diffusion coefficient in the liquid and will be therefore much higher than of dry GBs. Thus, in the regime $\eta /R \ll |\Delta | \ll 1$, the pre-melted GB dynamics corresponds to a classical curvature-driven GB dynamics with an enhanced mobility. Therefore, further investigations should focus on determining the structure of the triple junction, i.e. the structure of the liquid pocket, in these out-of-equilibrium conditions, and especially the deviation from the perfect $2\pi /3$ angles present at equilibrium. Further investigations should also address the question of the existence of out-of-equilibrium steady-state situations.

## Discussion

For our premelting problem, the phase field model is used in a way at odds with the usual philosophy of phase field theories, for example describing the pattern formation during solidification. Within the latter, the phase fields are functions that indicate which state the system presents locally. This state belongs to a discrete set, corresponding to the different stable or metastable phases under consideration. To each state corresponds a value of the phase fields, and the locus of their variations defines the interfaces. The interfaces’ width $\eta$, much smaller than the length scale of the pattern and the diffusion length in the case of diffusion-controlled processes, is considered as a purely numerical parameter upon which no outcome of the simulation should depend. In this respect, the bulk of the different phases and the interfaces between them are well separated and identified. The interface width $\eta$ should however be handled with care owing to its influence on the interface boundary conditions that the phase field model reproduces. Indeed, when the phase field model is recast into its corresponding sharp interface model using the so-called ’thin-interface limit’^39^, the interface boundary conditions depend on the equilibrium profiles of the phase fields and thus on $\eta$. The thin-interface limit is relevant at low enough driving forces, corresponding to linear kinetics obeying Onsager out-of-equilibrium thermodynamics^43^. Then, one is able to compare quantitatively the phase field results and the sharp-interface results, for example the growth velocity and the interfaces’ shape.

In opposition, for our premelting problem, the characteristic length scale of the liquid layer is $\eta$, and therefore no separation of length scales holds. The bulk of the liquid phase and the solid–liquid interfaces cannot be identified separately, and the liquid layer corresponds to a multi-phase region where at least two phase fields do not vanish. Moreover a physical meaning is attributed to the profile of the liquid phase field $\phi _3$. For example, its maximum, that varies continuously with temperature according to Eq. (9), is denoted as the ’degree of disorder’.

The premelting dynamics that we have simulated is, to a large extent, governed by the coupling of the phase fields in the multi-phase region and a special care should thus be taken in order to discretize the phase fields’ variations faithfully. As mentioned before, we had to use a grid step 75 times smaller than $\eta$ in order to reach a good precision on the conservation of energy. This is a tremendous increase in the number of grid points used to discretize the interface compared to usual studies, for example in solidification. We are not aware of any example for which such a resolution was needed. This necessity restricts the size of the domains that we were able to simulate. As an illustration, since we had to use very elongated simulation boxes in the *z*-direction in order to quantitatively reproduce the analytical results for $\Delta _{-\infty }$, we had to choose relatively small values of $\lambda$, i.e. $4\pi$ in the simulations presented in this article.

As we have seen previously, using a small $\lambda$ promotes premelting against melting, which occurs only when a certain overheating of the dry GB is exceeded, i.e. for $0<\Delta _\infty ^{(2)}<\Delta _\infty$. In Refs.^35,37^, melting along a dry GB was studied using a sharp-interface approach. A crucial ingredient of the theory is the contact angle at the triple junction. In^37^, only the macroscopic contact angle $\theta _\infty$ provided by Young’s law is considered. As mentioned before, it vanishes in our case $\sigma ^*>2$, and then, the steady-state growth velocity diverges. In Ref.^35^, a disjoining potential with an exponentially decreasing magnitude and a microscopic contact angle $\theta _0$ were introduced. As mentioned in the previous section, $\theta _0$ is related to the macroscopic angle and $\sigma ^*$ through $\theta _0^2 = \theta _\infty ^2 + \sigma ^*-2$, yielding a finite $\theta _0^2 = \sigma ^*-2$ when $\theta _\infty =0$. This regularization allows the velocity to become finite because, in the scaling laws inherited from the theory in^37^, $\theta _\infty$ is replaced by $\theta _0$ in^35^.

In both theories, the velocity is inversely proportional to $\sigma _{sl}$ for a given contact angle. This result opposes our simulations’ suggestion that the velocity is proportional to $\sigma _{sl}$ for a fixed $\sigma ^*$. This opposition may be apprehended when noting that the driving force for premelting is the reduction of interface energy parametrized by $\sigma _{gb}-2\sigma _{sl} = (\sigma ^*-2)\sigma _{sl}$, while the driving force for melting is the reduction of bulk free energy due to the stabilization of the liquid phase above $T_m$. It would be interesting to investigate the transition from an interface-energy-dominated driving force to a bulk-energy-dominated one with phase field simulations. The theories of melting in^35,37^ that yield a velocity inversely proportional to $\sigma _{sl}$ are derived for an infinite system in the direction perpendicular to growth (this is numerically possible because the Green’s function method that is used allows to eliminate the diffusion field and reduces to a search for the interface shape). As a consequence of energy conservation, the interface shape then assumes Ivantsov parabolic asymptotics far behind the tip^44^. The distance between the solid–liquid interfaces thus diverges, and the disjoining potential vanishes. Within our analysis of the phase field model, we have seen that, in a channel also, the disjoining potential vanishes in the equilibrated region far behind the tip when $\lambda >\lambda _c$. However, setting $\lambda$ only slightly beyond $\lambda _c$ does seem appropriate in order to reach a regime where the driving force is dominated by the reduction of bulk free energy. It seems indeed natural to suppose that this regime holds when the width of the liquid film is much larger than the spatial range on which structural forces act, i.e. when the liquid film is much larger than $\eta$. Then conservation of energy in the channel tells us that the width of the liquid film in the equilibrated region far behind the tip is approximately given by $\lambda \Delta _\infty$ (especially when ${\tilde{L}}$ is large). Thus, for a study of the premelting to melting transition under the close-to-equilibrium conditions assumed in Refs.^35,37^ ($\Delta _\infty \ll 1$), we need a simulation box such that $\lambda \gg \eta /\Delta _\infty \gg \eta$. In view of the extremely fine discretization required for an accurate realization of the complex coupling of the phase fields in the multi-phase region mentioned above, we understand that the investigation using phase field simulations of the cross-over between the regimes of bulk-energy-dominated and interface-energy-dominated driving force is rather challenging. The difficulty comes, of course, from the multi-scale nature of the problem. Of course, the multi-scale nature of the problem represents also a challenge numerically for the simulation of polycrystalline evolutions.

## Conclusion

A multi-phase field model with obstacle potentials was used to study, for the first time, the dynamics of grain boundary premelting. In the model, the disjoining potential describes structural forces on the scale of the interface width (on the atomic scale in a sharp-interface approach) and decreases monotonically with the distance from the solid–liquid interfaces. The model was used to simulate the steady-state growth of a liquid layer along a dry GB in an insulated channel and the evolution of a pre-melted polycrystalline microstructure.

Our results show that a transition exists from a premelting transformation, which produces a pre-melted equilibrium state with a finite disjoining potential energy, to a melting transformation, which produces a macroscopic solid–liquid equilibrium with a vanishing disjoining potential energy. The results also reveal that, due to energy conservation, confinement, which is linked to grain size (or channel width), promotes premelting against melting, implying that a certain overheating of dry GBs above the bulk melting temperature should be exceeded for melting to occur; that overheating is found to be inversly proportional to grain size. Conversely, for a given overheating, melting occurs only at large enough grain sizes. Our computational results also show that premelting dynamics is governed by the reduction of the interface energy, with a velocity proportional to $\sigma _{gb} - 2\sigma _{sl}$.

We found that a polycrystalline equilibrium exists in which the triple junction takes the form of a liquid pocket with a macroscopic size. Realizing the presence of that equilibrium allowed us to gain novel insights into the evolution of pre-melted polycrystalline microstructures. That evolution consists of two stages: in the first stage, the liquid premelts the dry GBs, and then, in the second stage, the fully pre-melted GBs evolve. Due to the presence of the polycrystalline equilibrium, the disjoining potential plays a crucial role not only during the first stage, but also during the second one. For example, it allows the grains to have morphologies that, in the absence of the disjoining potential, would be energetically unfavorable. In addition, we find that, again due to the presence of the polycrystalline equilibrium, if grains within a premelted microstructure are large enough, in weakly out-of-equilibrium conditions, the dynamics may be recast into the well-known curvature-driven dynamic of dry GBs, with a mobility that is enhanced by a factor proportional to the ratio of liquid to solid diffusion coefficients.

An interesting future study is investigating melting along a dry GB when $\sigma _{gb} -2 \sigma _{sl}>0$, and especially the transition between the regime driven by the reduction of interface energy, as in our simulations, and the one driven by the reduction of bulk free energy. Another interesting future study concerns the influence of the disjoining potential on the contact angles at the triple junction where the dry GB meets the two solid–liquid interfaces. Concerning the evolution of pre-melted polycrystalline microstructures, the perspective is to study the phenomenology arising from, as we have shown, the existence of three relevant length scales, i.e. the width of the liquid layer, the size of the liquid pocket at the pre-melted triple junction and the grain size.

## Methods

The governing equations of the model can be outlined as follows. The total free energy reads

19 $$\begin{aligned} F = \int _V f(\phi _1, \phi _2, \phi _3, T)\;dV, \end{aligned}$$

where the local free energy density that depends on the spatial distribution of the three phase fields and the temperature reads

$$\begin{aligned}&f(\phi _1, \phi _2, \phi _3, T) = L \phi _3^2(3-2\phi _3) \frac{T_m-T}{T_m} \nonumber \\&- \frac{4 \sigma _{sl} }{\eta } \left[ \frac{\eta ^2}{\pi ^2} ({\varvec{\nabla }}\phi _1 \cdot {\varvec{\nabla }}\phi _3 + {\varvec{\nabla }}\phi _2 \cdot {\varvec{\nabla }}\phi _3 + \sigma ^* {\varvec{\nabla }}\phi _1 \cdot {\varvec{\nabla }}\phi _2) - (\phi _1 \phi _3 + \phi _2 \phi _3 + \sigma ^* \phi _1 \phi _2) \right] \end{aligned}$$

where $\sigma ^* = \sigma _{gb}/\sigma _{sl}$ tunes the tendency to premelting that occurs when $\sigma ^*>2$.

In a dimensionless form and expressing lengths in units of $\eta /(2\pi )$, we have

20 $$\begin{aligned} {\tilde{F}} = \int _{{\tilde{V}}} {\tilde{f}}(\phi _1, \phi _2, \phi _3, T) d{\tilde{V}}, \end{aligned}$$

with

21 $$\begin{aligned}&{\tilde{f}} = \frac{f \eta }{4 \sigma _{sl}} = - \phi _3 u - 4\left[ {\tilde{{\varvec{\nabla }}}} \phi _1 \cdot {\tilde{{\varvec{\nabla }}}} \phi _3 + {\tilde{{\varvec{\nabla }}}} \phi _2 \cdot {\tilde{{\varvec{\nabla }}}} \phi _3 + \sigma ^* {\tilde{{\varvec{\nabla }}}} \phi _1 \cdot {\tilde{{\varvec{\nabla }}}} \phi _2 \right] + \phi _1 \phi _3 + \phi _2 \phi _3 + \sigma ^* \phi _1 \phi _2 \end{aligned}$$

22 $$\begin{aligned}&\text {with } u = \frac{ L \eta (T-T_m) }{4 \sigma _{sl} T_m}, \tilde{\varvec{\nabla }}= \frac{\eta {\varvec{\nabla }}}{2\pi } \end{aligned}$$

The phase fields evolution equations are given, when the three phases are present (i.e. $\phi _1 \phi _2 \phi _3 \ne 0$), by

23 $$\begin{aligned} {\dot{\phi }}_i = \frac{M_{ij}}{3} \left( \frac{\delta {\tilde{F}}}{\delta \phi _j} - \frac{\delta {\tilde{F}}}{\delta \phi _i} \right) + \frac{M_{ik}}{3} \left( \frac{\delta {\tilde{F}}}{\delta \phi _k} - \frac{\delta {\tilde{F}}}{\delta \phi _i} \right) \end{aligned}$$

with $(i,j,k) = (1,2,3), (2,3,1)$ or (3, 1, 2). When one of the phase field vanishes, for example $\phi _k=0$, then

24 $$\begin{aligned} {\dot{\phi }}_i = \frac{M_{ij}}{2} \left( \frac{\delta {\tilde{F}}}{\delta \phi _j} - \frac{\delta {\tilde{F}}}{\delta \phi _i} \right) . \end{aligned}$$

The functional derivatives are given by

$$\begin{aligned} \frac{\delta {\tilde{F}}}{\delta \phi _1} = \left( 1+4 \tilde{\varvec{\nabla }}^2 \right) (\sigma ^* \phi _2 + \phi _3); \; \frac{\delta {\tilde{F}}}{\delta \phi _2} = \left( 1+4 {\tilde{{\varvec{\nabla }}}}^2 \right) (\sigma ^* \phi _1 + \phi _3); \; \frac{\delta \tilde{F}}{\delta \phi _3} =- 6 \phi _3 (1-\phi _3 )u + \left( 1+4 \tilde{\varvec{\nabla }}^2 \right) (\phi _1 + \phi _2) \end{aligned}$$

On the other hand, the temperature equation reads

25 $$\begin{aligned} \dot{u} = \frac{D }{\eta ^2/(2\pi )^2} {\tilde{{\varvec{\nabla }}}}^2 u - 6 \phi _3(1-\phi _3) \frac{{\tilde{L}}}{S} \; {\dot{\phi }}_3 \end{aligned}$$

where *D* is the diffusivity, taken constant throughout the whole simulation domain, and with ${\tilde{L}} = L\eta /\sigma _{sl}$ and $S = 4 c_p T_m/L$.

## Supplementary information

Supplementary Video.

## Data Availability

The data that support the findings of this study are available on request from the corresponding author [GB].
